# Establishment of Organoids From Human Epithelioid Sarcoma With the Air-Liquid Interface Organoid Cultures

**DOI:** 10.3389/fonc.2022.893592

**Published:** 2022-05-23

**Authors:** Toru Wakamatsu, Hisataka Ogawa, Keiichi Yoshida, Yukiko Matsuoka, Kazuko Shizuma, Yoshinori Imura, Hironari Tamiya, Sho Nakai, Toshinari Yagi, Shigenori Nagata, Yoshihiro Yui, Satoru Sasagawa, Satoshi Takenaka

**Affiliations:** ^1^ Department of Musculoskeletal Oncology Service, Osaka International Cancer Institute, Osaka, Japan; ^2^ Department of Orthopaedic Surgery, Osaka University Graduate School of Medicine, Osaka, Japan; ^3^ Nitto joint Research Department for Nucleic Acid Medicine, Research Center, Osaka International Cancer Institute, Osaka, Japan; ^4^ Department of Gastroenterological Surgery, Osaka University Graduate School of Medicine, Osaka, Japan; ^5^ Next-generation Precision Medicine Research Center, Osaka International Cancer Institute, Osaka, Japan; ^6^ Department of Diagnostic Pathology and Cytology, Osaka International Cancer Institute, Osaka, Japan; ^7^ Sarcoma Treatment Laboratory, Research Institute, Nozaki Tokushukai Hospital, Osaka, Japan; ^8^ Molecular Biology Laboratory, Research Institute, Nozaki Tokushukai Hospital, Osaka, Japan

**Keywords:** epithelioid sarcoma, organoid, air-liquid interface organoid culture method, xenograft, integrase interactor 1

## Abstract

**Background:**

Although biological resources are essential for basic and preclinical research in the oncological field, those of sarcoma are not sufficient for rapid development of the treatment. So far, some sarcoma cell lines have been established, however, the success rate was low and the established sarcoma types were frequently biased. Therefore, an efficient culture method is needed to determine the various types of sarcomas. Organoid culture is a 3-dimentional culture method that enables the recapitulation of the tumor microenvironment and the success rate reported is higher than the 2-dimentional culture. The purpose of this study was to report our newly established organoids from human epithelioid sarcoma using the air-liquid interface organoid culture method.

**Methods:**

We treated 2 patients with epithelioid sarcoma in our institute. The remaining sarcoma specimens after surgical resection were embedded in collagen type 1 gels according to the air-liquid interface organoid culture method. After serial passages, we xenografted the organoids to NOD-scid IL2Rgnull (NSG) mice. Using the developed tumors, we performed histological and genomic analyses to compare the similarities and differences with the original epithelioid sarcoma from the patient.

**Results:**

Organoids from the epithelioid sarcoma could be serially cultured and maintained in collagen type 1 gels for more than 3 passages. Developed orthotopic tumor xenografts were detected in the NSG mice. After the process was repeated severally, the patient derived organoid lines from the epithelioid sarcoma were established. The established organoids showed loss of integrase interactor 1 expression with polymerase chain reaction and immunohistochemical analyses. The xenografted organoids of the epithelioid sarcoma had histologically similar phenotypes with the original tumor and genetically resembled it to some degree.

**Conclusions:**

The present study demonstrated 2 novel established organoid models of epithelioid sarcoma, and our organoid models could be used to investigate the molecular pathogenesis and develop a novel treatment.

## Introduction

There are over 200 histological types of sarcomas. Sarcomas are classified as rare cancers, that is, cancers with an incidence of 6/100000 patients per year (1, 2). The prognosis of rare cancers, including sarcomas, is poorer than that of common cancers (3). One of the reasons is that the biological resources of sarcomas are not sufficient for advanced research (4, 5). Therefore, we established several types of sarcoma cell lines, including synovial sarcoma, Ewing sarcoma, clear cell sarcoma, CIC-rearranged sarcoma, and epithelioid sarcoma (EPS) (6–9). However, several histological types of sarcomas cell lines have not been established. Spheroid culture methods have been used to investigate the biological characters of some sarcomas (10, 11). However, an efficient treatment for sarcoma has not been developed, thus it is essential to develop an efficient culture method which could be applied for the various types of sarcomas.

In order to solve the problem, we focused on the 3-dimentional (3D) organoid culture methods that have been widely applied in various types of cancer (12–17). The culture method enables to mimic tumors in a living human body compared to adhesion culture (2D) and to recapitulate the tumor microenvironment and heterogeneity. In addition, the reported success rate of established tumor cell lines was higher than that of the 2D culture. The air liquid interface (ALI) organoid culture method is one of the organoid culture methods and it was first reported as the methodology for primary mouse intestinal culture allowing sustained intestinal proliferation and multilineage differentiation (18, 19). The ALI organoid culture method was applied to investigate the cancer organoid or tumor immune microenvironment (20, 21). In the past, sarcoma cells were cultured with other organoid culture methods using Matrigel, but it was unsuccessful. Instead, the ALI method, which uses collagen and enables the aeration and polarity of cells, was employed to establish the sarcoma organoids. Recently, a study on osteosarcoma organoids, but not soft tissue sarcomas including EPS, using the ALI method have been reported, thus the organoid culture methods are not widely applied for sarcoma (22, 23).

EPS is a relatively rare type of malignant mesenchymal neoplasm with both epithelial and mesenchymal cytomorphology and immunophenotype (24, 25). EPS occurs in various anatomic locations and young adults. There are two clinicopathological subtypes including the classic (or distal) form and proximal type (large cell). EPS is genetically characterized by loss of INI1, encoded by SMARCB1, that is one of the components of SWI/SNF complex (26, 27). EPSs have high local recurrent and metastatic potentials. The effective treatment of EPS including chemotherapy except for surgery has not been reported (28–32).

In this study, we reported 2 cases of EPS and the establishment of the patient derived organoids (PDO) from the EPS tumor. We developed 2 organoid derived xenograft (ODX) models from the derived EPS PDO and described their cytomorphology and immunophenotype features and genetics.

## Materials and Methods

### Patient 1 (OICI-EPS-0530)

**Figure 1:**
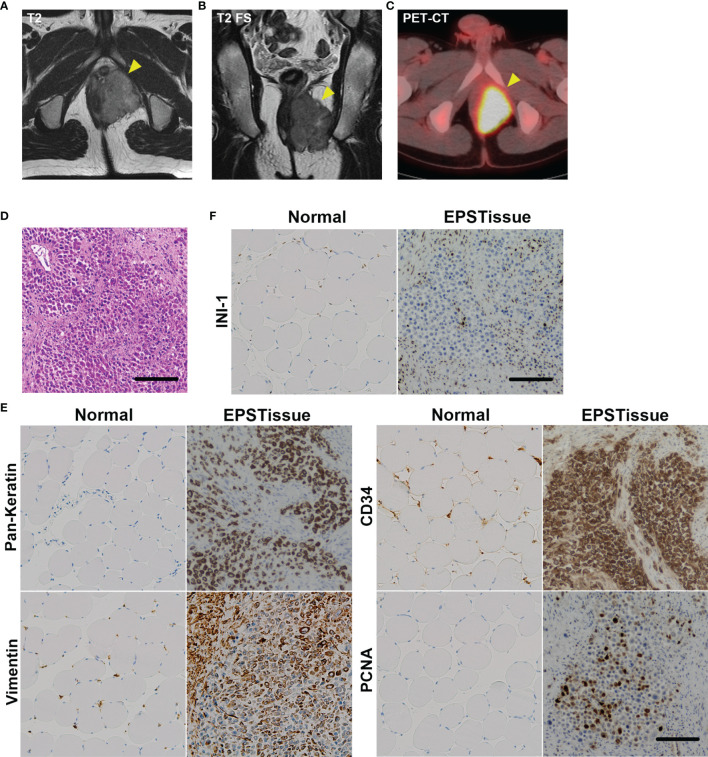
Clinical images of the EPS tumor in the left perineum of a 22-year-old man. **(A)** T2-weighted axial image of MRI. **(B)** T2-weighted fat-suppressed sequence (T2 FS) coronal image of MRI. **(C)** The axial image of positron emission tomography-computed tomography (PET-CT). **(D)** Histological appearance of the original tumor with H&E staining. **(E)** Immunohistochemical reactivity in the original tumor. Tumor cells were diffusely positive for AE1/AE3, vimentin and CD34, but negative for INI-1 **(F)**. MIB-1 labeling index was approximately 20%. Scale bars: 100μm.

The patient was a 22-year-old Japanese man that presented with a painful tumor measuring 5.3 cm that was located in the left perineum. Magnetic resonance imaging (MRI) and positron emission tomography were performed suspecting a malignant soft tissue tumor. Computed tomography revealed a non-metastatic lesion (**Figures 1A–C**). A needle biopsy was performed and revealed a distal type epithelioid sarcoma. The epithelioid cells with acidic vesicles and eccentric nucleus showed infiltrative growth (**Figure 1D**). Immunoreactivity for cytokeratin (AE1/AE3), vimentin, and CD34 was positive but integrase interactor 1 (INI-1) was negative for the tumor cells (**Figures 1E, F**). Wide resection of the bladder, prostate, penis, and rectum was performed, and creation of a stoma and urinary diversion were performed. After adjuvant radiotherapy with 66Gy/33fr, 3 cycles of adjuvant chemotherapy consisting of doxorubicin (60 mg/m^2^) and ifosfamide (10 g/m^2^) were administrated. The patient was in continuous disease-free (CDF) for more than 1 year.

### Patient 2 (OICI-EPS-0486)

The patient was a 50-year-old Japanese man that presented with a painful tumor measuring 11 cm that was located in the right proximal thigh. Magnetic resonance imaging (MRI) was performed suspecting a malignant soft tissue tumor. Computed tomography revealed a multiple subcutaneous metastatic lesions (**Supplementary Figure 3A**). A open biopsy was performed and revealed a distal type epithelioid sarcoma. The polygonal to spindle shaped cells with giant and polymorphic nucleus showed infiltrative growth with wide lesion of inflammation and necrosis (**Supplementary Figure 4A** left). Immunoreactivity for pan-keratin, vimentin was positive and CD34 was faintly stained in tumor cells. But integrase interactor 1 (INI-1) was negative for the tumor cells (**Supplementary Figures 4B, C**). Wide resection of the tumor and proximal femur was performed, after that proximal femoral replacement was performed. After the surgery we planed adjuvant chemotherapy, but the patient did not want chemotherapy and chose the best supportive care. The patient was in dead of disease (DOD) 2 months after surgery.

### Culture Medium

The basal medium consisted of Advanced DMEM/F12 (Thermo fisher Scientific) supplemented with HEPES (10 mM, Thermo fisher Scientific), GlutaMAX (1x, Thermo fisher Scientific), and Penicillin-Streptomycin-Glutamine (1x, Thermo fisher Scientific).

The organoid culture medium, which was used for the organoid culture of human prostate cancer, consisted of the basal medium supplemented with nicotinamide (10 mM, Sigma), N-acetylcysteine (1 mM, Wako), A-83-01 (500 nM, Wako), B-27 (1x, Gibco), recombinant human EGF (50 ng/mL, Sigma), gastrin I human (10 nM, Peprotech), recombinant human Noggin (100 ng/mL, Peprotech), recombinant human R-spondin 1 protein (500 ng/mL, R&D), SB-202190 (10 μM, Chemscene), and 10% Afamin/Wnt3a CM (MBL) (**Table S1**).

### Air-Liquid Interface (ALI) Organoid Cultures

We prepared the collagen gel matrix (Cellmatrix type I-A, Nitta Gelatin Inc.) according to the protocol of organoid culture procedure as previously described (26, 27). In detail, on ice, we mixed Cellmatrix I-A with 10 X concentrated sterile culture medium (Ham’s F-12) and sterile reconstitution buffer (2.2g NaHCO3 in 100ml of 0.05N NaOH and 200mM HEPES) at a ration of 8:1:1. During the mixing, the creation of bubbles was avoided. The mixed solution was kept on ice (at 4°C) to prevent gel formation.

For the preparation of the culture dish, Millicell culture plate inserts (PICM03050, Millicell-CM, Millipore), with permeable and membranous bottoms, were placed in a 60 mm tissue culture dish. To creat the bottom layer, 1 ml of the prepared reconstituted collagen solution was added to the inserts under sterile conditions. The culture dish with the inserts was incubated until solidification in an incubator at 37°C for 30 min (**Supplementary Figures 1A, B**). During the incubation, tissue preparation was performed as described below.

### Tissue Preparations

The remaining surgically resected tissue was used for the experiments following the guidelines of the Institutional Review Board for Clinical Research after obtaining the patient informed consent. After measuring the weight of the tumor tissues with scales, the tumor tissues were washed four times with iced phosphate-buffered saline (PBS) in 50 ml conical tubes by tipping over. After that, they were minced with sterilized scissors and scalpel on the 60 mm dish for 15 minutes at room temperature. The minced tumor tissues were collected with 3 ml of Dulbecco’s Modified Eagle’s Medium (DMEM; GIBCO, Grand Island, NY, USA) using a 5-ml pipette into a 10 ml conical tube and centrifuged at 600g for 5 min. The cell suspensions were incubated with 50 μg/ml of Liberase TH (Sigma-Aldrich, St. Louis, MO, USA) for 15 min at 37°C. The supernatant was discarded, and the cells were incubated in fetal bovine serum (FBS; Sigma) at 37°C for 15min to inactivate Liberase TH. The cells were suspended into 30 ml DMEM and passed through a 1 mm nylon mesh (Becton Dickinson Falcon, Franklin Lakes, NJ, USA) to remove the debris when needed. After four times washing with basal medium, the prepared cells were suspended into the upper layer of the prepared collagen and 1.5 ml of organoid culture medium were added into the culture dish on the outside insert (**Supplementary Figures 1A, B**). The organoids were cultured at 37°C with 95% air, 5% CO2, and 100% humidity. The organoid culture medium was changed twice a week.

### Organoid Passage

The organoids were passaged after two to four weeks after the organoids culture. The upper and bottom layers were transferred into another 60 mm culture dish with tweezers and the organoids with the layers of collagen were minced with sterilized scissors and scalpel on the dish for 15 minutes at room temperature. After incubation with 50 μg/ml of Liberase TH for 15 min at 37°C, the cells were centrifuged at 600g for 5 min and supernatant was discarded, and they were incubated in FBS at 37°C for 15min to inactivate Liberase TH. After two times washing with basal medium, the prepared cells were cultured in the upper layer of the prepared collagen at 37°C with 95% air, 5% CO2, and 100% humidity.

### Organoid Freeze Preservation and Reculture

The minced organoids obtained during the passage were frozen in Stem Cell Banker (Amsbio ZENOAQ, 11890) and stored in -80°C freezer. During reculture of the preserved organoids, the cryotube was warmed in water at 37°C for 5 min. After washing twice with the basal medium, the prepared cells were cultured in the upper layer of the prepared collagen at 37°C with 95% air, 5% CO2, and 100% humidity.

### Establishment of Novel PDO From an EPS

**Figure 2:**
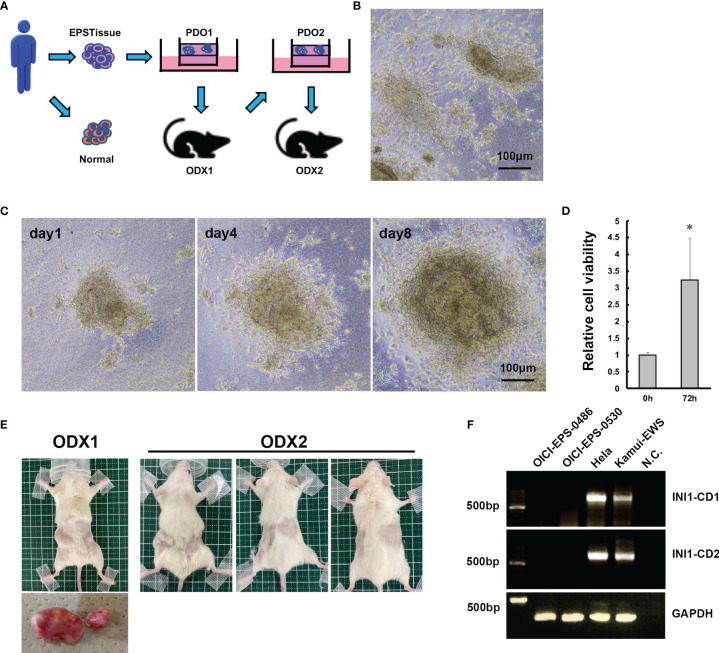
Establishment of PDO and ODX models of OICI-EPS-0530. **(A)** The scheme of the establishment of PDO and ODX. **(B)** The morphology of OICI-EPS-0530 organoids under ALI organoid culture by phase-contrast microscopy. **(C)** Growing organoid images of OICI-EPS-0530 at day 1, day 4 and day 8. Scale bars: 100μm. **(D)** Relative organoids viability from 0 h to 72 h. (n = 6; *P < 0.05). **(E)** Developed tumors in NSG mice of ODX-1 (n=1) and ODX-2 (n=3) from PDO of OICI-EPS-0530. **(F)** RT-PCR with the primer set in INI1-CD1 and INI1-CD2. No band was present for the negative control (N.C.) of distilled water. Hela and Kamui-EWS (Ewing sarcoma cell line established in our institute) were demonstrated for positive control.

EPS tumor tissues were prepared using the above method, and the tumor cells were cultured with ALI organoid culture. After several serial passages, the cultured cells were xenografted in a NOD-scid IL2Rgnull (NSG) mouse. The developed tumor was resected from the mouse, and the same steps of tissue preparation were performed as described above. The ALI organoid culture was repeated for the serial passages and xenograft in NSG mice. When the tumor developed, the PDO was established from EPS (**Figure 2A**).

### RNA Isolation and Reverse Transcription Polymerase Chain Reaction (RT-PCR)

The total RNA was extracted using RNeasy Plus Universal Mini Kit (Qiagen) according to the manufacturer’s protocol, and the determined purity was A260 to A280. For cDNA synthesis, the Superscript IV VILO (Invitrogen) was used according to the manufacturer’s instructions. RT-PCR was performed with PrimeSTAR MAX DNA polymerase (Takara). The primers, designed according to Ref. 32 for *INI1*, are listed in the supplementary material (**Table S2**).

### DNA Isolation

The total DNA was extracted using DNeasy Blood & Tissue Kit (Qiagen) according to the manufacturer’s protocol.

### Sequence Analysis

*EWS-FLI1* cDNA or DNA was identified by polymerase chain reaction (PCR) using a primer set of *EWS-FLI1* or genomic*EWS-FLI1* (**Table S2**). For sequence analysis, the reverse-transcriptase (RT) PCR-amplified *EWS-FLI1* cDNA or genomic DNA fragments were analyzed on 1.5% agarose gels, purified using a Qiagen gel extraction kit (Qiagen, Hilden, Germany), and directly sequenced in Eurofins Genomics with forward or reverse primers mentioned above. BLAST software (http://blast.ncbi.nlm.nih.gov/Blast.cgi) was used for computer analysis of sequence data.

### Immunohistochemistry

Immunohistochemical studies were performed to clarify whether the phenotype of the cultured organoids and developed tumors matched with that of the original tumors. The specimens of the tumors and organoids were fixed in 10% neutral, buffered with formalin, embedded in paraffin, sectioned at 4 μm thickness, and stained with hematoxylin and eosin. The paraffin embedded sections were deparaffinized and dehydrated. The antigens were retrieved at 95°C for 15 min in PH6.0 citric acid buffer. After blocking the endogenous peroxidase activity for 15 min with VECTASTAIN Elite ABC Universal PLUS Kit Peroxidase (VECTOR PK-8200), the sections were reacted for 1 h with 2.5% Normal Horse Serum at room temperature to prevent nonspecific binding. They were then incubated overnight with primary antibody in a blocking solution at 4°C. After six subsequent PBS washings, the appropriate prediluted biotinylated secondary antibodies (1:500, BD Biosciences) were added for 30 min. The sections were incubated for 30 minutes with VECTASTAIN Elite ABC Reagent, following by DAB staining (ImmPACT DAB EqV solutions) until the appropriate stain intensity develops (1-2 min). The slides were then washed and counterstained with Gill No. 3 hematoxylin (Sigma) for 45 s. As a negative control, staining was carried out in the absence of primary antibodies. The histological analyses were checked by a pathologist in our institute. The primary antibodies against Pan-Keratin (C11) (1:500, Cell Signaling Technology, USA), Vimentin (D21H3) Xp (1:200, Cell Signaling Technology, USA), CD34 (B-6) sc-74499 (1:100, Santa Cruz), INI-1 (D8M1X) (1:1000, Cell Signaling Technology, USA), and PCNA (D3H8P) XP (1:5000, Cell Signaling Technology, USA) were used.

### Organoid Proliferation Assay

Cultured EPS organoids or the developed tumor in the mouse were used for the proliferation assay. Cell suspension was prepared according to the organoid passage or tissue preparation procedures. The collagen gel matrix was prepared according to the protocol of the organoid culture procedure. We scaled down the experiment because the prepared cell number and repetition of the experiments (**Figures 6B, C**) were limited. For the preparation of the culture dish, Millicell culture plate inserts (PICM 01250, Millicell-CM, Millipore) were placed in a culture plate with 24 wells (Thermo). In order to form the bottom layer, 150 μl of the prepared reconstituted collagen solution was added to the inserts under sterile conditions. The culture plate with the inserts was incubated until solidification in an incubator at 37°C for 30 min. During the incubation, the steps of tissue preparation described above were performed. The prepared cells were counted and cultured in the upper layer of the prepared 150 μl of collagen with 200 μl of organoid culture medium at 37°C with 95% air, 5% CO2, and 100% humidity for 72h.

Cell counting Kit-8 assays were performed to measure the cell viability. We measured the viability of the cells at 0 h and 72 h after the start of organoid culture. The ratio of cell viability was calculated.

3-(4,5-Dimethyl-2-thiazolyl)-2,5-diphenyltetrazolium Bromide (MTT) assays were used to detect live organoids 72 hours after treatments with doxorubicin (Wako) at concentrations of 0.015–15M or paclitaxel (Wako) at concentrations of 0.04-4μM. We detected the live organoids by adding 100 μl of 5 mg/ml MTT solution in PBS and incubated for 4 h. Only live organoids could be detected as blue spots and counted the spot areas with ImageJ according to the manufacturer’s protocol.

### Lentivirus Generation and Infection

The lentiviral packaging mixture (pLP1, pLP2, and pLP/VSVG) were purchased from Invitrogen. The GFP expression vector, pLentiCMV GFP Puro (658–5), and lentivirus packaging mixture were co-transfected into 293FT cells using PEI-MAX instead of Lipofectamine 2000 according to Invitrogen’s instruction. The lentivirus containing supernatants were collected 48h after transfection and used to infect the target cells. The target OICI-EPS-0530 cells were cultured in an adherent culture dish prepared from the organoids. Furthermore, 24h after infection, the culture medium was replaced with fresh organoid culture medium. The cells were then cultured with ALI organoid culture method and applied to each assay.

### Mouse Studies

All the animal experiments were approved by the institutional committee of animal care of the Osaka International Cancer Institute, and all the animals were euthanized with diethyl ether at the end of the experiments. The 6-week-old NSG mice were purchased from Japan SLC (Shizuoka, Japan). The upper and bottom layers were transferred into another 60 mm culture dish with tweezers and incubated the organoids with 50 μg/ml of Liberase TH for 15 min at 37°C. The cells were centrifuged at 600g for 5 min and the supernatant was discarded. The organoids were suspended into 50 μl PBS. After mixing with 50 μl of growth factor reduced Matrigel (BD Biosciences), the mixed solution was kept on ice (at 4°C) until xenograft. Small incisions on both sides of the back in the NSG mice were made, and the prepared organoid suspension were injected using a syringe without a needle. The incisions were stiched thereafter. The mice were inspected once a week and were sacrificed when the total tumor burden reached 1-1.5 cm^3^. The tumors were then resected and used for organoid culture, RNA collections, and immunohistochemical studies.

### RNAseq

The total RNA of samples were sent to Seibutsu Giken Inc (Kanagawa, Japan) for next generation sequencing (NGS). The concentrations of the RNA samples were measured using the Quantus Fluorometer and Quanti Fluor RNA system (Promega). Quality check of the RNA was performed with 5200 Fragment Analyzer System and Agilent HS RNA Kit (Agilent Technologies). The NGS library was prepared by MGIEasy RNA Directional Library Prep Set (MGI Tech Co Ltd) according to the manufacturer’s protocol. The concentrations of the library were measured with Synergy LX and Quanti Fluor dsDNA System. Quality check of the library was performed with Fragment Analyzer and dsDNA915ReagentKit (Advanced Analytical Technologies). A circular DNA was made from the library by MGIEasy Circularization Kit (MGI Tech Co Ltd). DNA Nanoball (DNB) was made with DNBSEQG 400 RS High throughput Sequencing Kit (MGI Tech Co Ltd) according to the manufacturer’s protocol. Sequencing was performed with DNBSEQ-G400 for at least 2 × 100 bp read. The adapters and primers from the delivered fastq files were removed using cutadapt (ver. 1.9.1). Short read sequences under 20 and low-quality score reads (under 40) were removed with a sickle (ver. 1.33). HISAT2 (ver. 2.2.1) was used to read the alignment and mapping of the GRCh38 reference genomes. After reading and writing the alignment data in the SAM and BAM formats with the SAMtools, the data were sorted and indexed. The read counts per gene were obtained using the featureCounts (ver. 2.0.0). The relative expression level of each gene was normalized by the RPKM (Reads per Kilobase Million) and Transcripts Per Million methods. We analyzed and visualized the genetic characteristics and transcriptome profiling with iDEP and RaNAseq (33, 34). The presence of fusion gene was analyzed by Seibutsu Giken Inc.

### Statistical Analysis

Statistical significance was set at P < 0.05. Statistical analyses were performed using the Student’s t test. Ethical approval for this study was obtained from the institutional review board of our institute.

## Results

### Organoid’s Morphology and Growth of OICI-EPS-0530 and OICI-EPS-0486 With ALI Organoid Culture Method

We cultured the EPS cells using the ALI organoid culture method and labeled the organoid lines as OICI-EPS-0530 and OICI-EPS-0486. The growth with time was stable in the serial passages. The organoid morphology was distorted into a spherical shape and small clumps of cells, and disperse cells were observed (**Figure 2B** and **Supplementary Figure 3B**). The size of the OICI-EPS-0530 and OICI-EPS-0486 organoid increased in a short period and their viability increased by approximately 3.3-fold and 2.3-fold in 72 h, respectively (**Figures 2C, D** and **Supplementary Figures 3B, C**). In addition, the organoids were easily broken during the pipetting phase of the passage procedures, implying that the cell-cell adhesion in the organoids was not strong.

### Tumorigenicity of OICI-EPS-0530 and OICI-0486 Organoids in NSG Mice

Thirty-six days after xenograft of the OICI-EPS-0530 organoids (PDO1) into a NSG mouse, the tumor developed (ODX1) (**Figure 2E**). The tumors were then resected, minced according to the tissue preparations, and cultured using the ALI organoid methods (PDO2). These replated organoids showed similar morphological features to the original organoids from the patient tumor tissues. Fifty-three days after xenograft of the re-cultured organoids into 3 NSG mice, the tumors developed in all the xenografted mice (ODX2) (**Figure 2E**). This means that the OICI-EPS-0530 had high tumorigenesis. The cells prepared from the ODX2 were able to grow in the ALI organoid cultures. In the same manner, 27 days after xenograft of the OICI-EPS-0486 organoids (PDO) into a NSG mouse, the tumor developed (ODX) (**Supplementary Figure 3D**).With the RT-PCR, no INI-1 expression was detected in the OICI-EPS-0530 tumors similar to OICI-EPS-0486 (**Supplementary Figure 3A**), in contrast to Hela and an Ewing sarcoma cell line, Kamui-EWS, which was used as a positive control (**Figure 2F** and **Supplementary Figures 2A–E**).

### Morphologic Characteristics of OICI-EPS-0530 and OICI-EPS-0486

**Figure 3:**
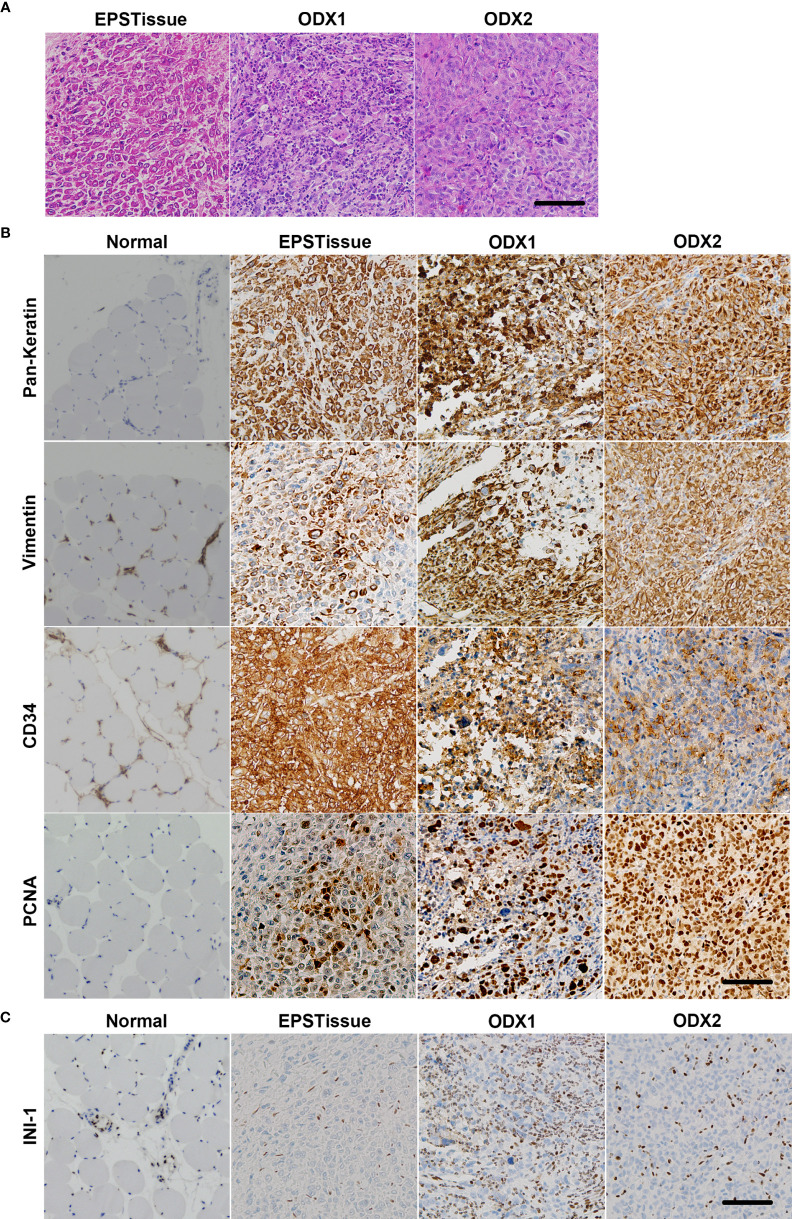
Comparing the microscopic features of the patient’s original tumor (EPSTissue), and ODXs of OICI-EPS-0530. **(A)** The histological appearance of the EPSTissue and ODXs of OICI-EPS-0530 with H&E staining. **(B, C)** Immunohistochemical reactivity in EPSTissue and ODXs of OICI-EPS-0530. Tumor cells were diffusely positive for pan-keratin, vimentin, CD34 and PCNA **(B)**. But tumor cells were negative for INI-1 **(C)**. Scale bars: 100μm.

**Figure 4:**
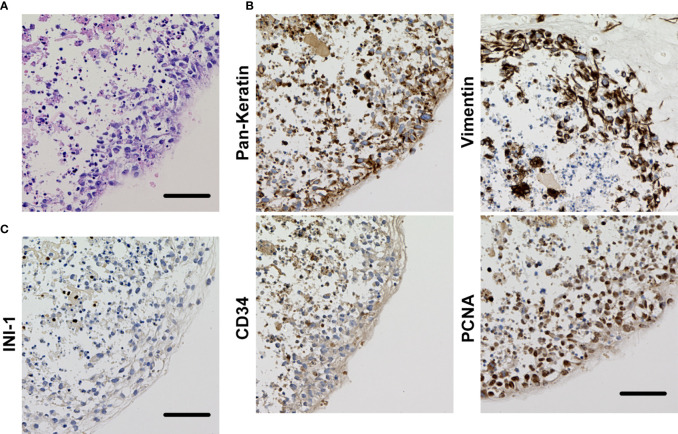
**(A)** Histological appearance of the organoid of OICI-EPS-0530 with H&E staining. **(B)** Immunohistochemical reactivity in the organoid of OICI-EPS-0530. Tumor cells were diffusely positive for AE1/AE3, vimentin, CD34 and PCNA. **(C)** Tumor cells were negative for INI-1. Scale bars: 100μm.

Hematoxylin and eosin staining of the OICI-EPS-0530 xenograft tumors (ODX1 and ODX2) demonstrated a heterogeneous histological appearance resembling the original tumor (**Figure 3A**). They showed a multinodular proliferation of eosinophilic epithelioid and spindle-shaped tumor cells. The immunoreactivity for pan-keratin, vimentin, CD34 and PCNA was positive, but INI-1 was negative in the ODX1 and ODX2 tumors similar to the original EPS tumor tissue (**Figures 3B, C**). In addition, hematoxylin and eosin staining of the OICI-EPS-0530 organoids showed distorted spherical morphology and peripheral cells of the organoids were epithelioid and spindle-shaped tumor cells (**Figure 4A**). The immunoreactivity for pan-keratin, vimentin, CD34 and PCNA was positive, but INI-1 was negative in the organoids similar to the original EPS tumor tissue and xenograft tumors (**Figures 4B, C**). Almost similar results were obtained from OICI-EPS-0486 except for faint staining of CD34 (**Supplementary Figure 4**). These data demonstrated that the morphological characteristics of the OICI-EPS-0530 and OICI-EPS-0486 were preserved during organoid culture and xenograft.

### Genetic Characteristics and Transcriptome Profiling in OICI-EPS-0530

**Figure 5:**
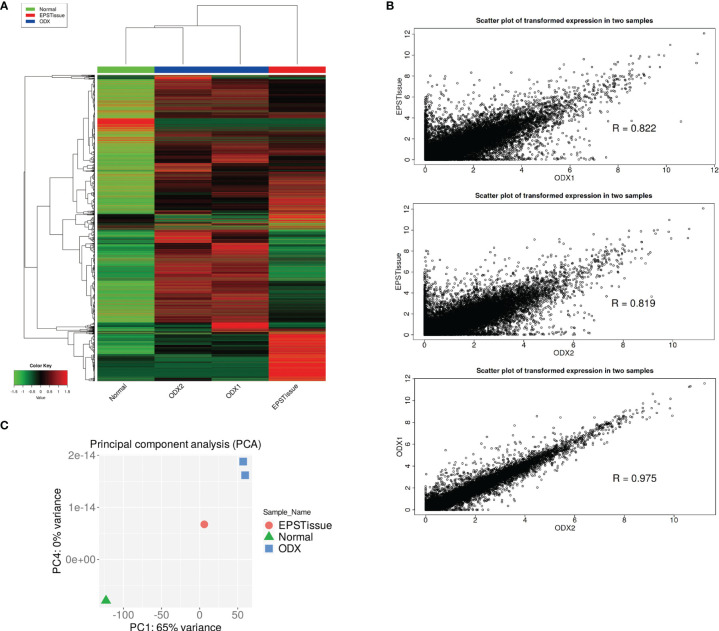
**(A)** Heatmap showing the gene expression profiling (9600 genes) of normal tissue (Normal), original EPS tumor (EPSTissue) and OICI-EPS-0530 tumors developed in NSG mice (ODX1 or ODX2). **(B)** Scatter plots showing the correlation of gene expression between OICI-EPS-0530 tumors (ODX1 or ODX2) and EPSTissue. R represents the correlation coefficient. **(C)** Principal component analysis (PCA) between Normal, EPSTissue and ODX1 and ODX2.

We conducted RNA-seq of paired tumors and normal samples from patients with EPS and developed the tumors in mice (ODX1 and ODX2). The gene expressions of each sample were visualized in heatmap, scatter plot and principal component analysis (**Figures 5A–C**). The gene expression profiles of the normal tissue did not correlate with those of the original tumors, ODX1 and ODX2 (**Supplementary Figures 3A–C**). The gene profile of the original tumor showed moderate correlations with the profiles of ODX1 and ODX2. The gene profiles between ODX1 and ODX2 strongly correlated. These results suggested that the gene expression signatures of the OICI-EPS-0530 tumors were not completely identical to those of the primary EPS tumor. In contrast, the gene expression signatures were preserved in the PDOs and ODXs. No fusion gene was detected in all RNA samples.

The transcriptome analysis showed that 10116 and 1732 genes were upregulated and downregulated in EPS ODX compared to the normal tissue at fold changes > 2, respectively (**Supplementary Figure 4A** and **Tables S3****, and** **S4**). We performed Kmeans Enrichment analyses for gene functional and ontological characterization (**Supplementary Figure 4B** and **Tables S5****,** **S6**). The gene expressions were clustered in four groups and cluster C was enriched in both the original EPS tumor and ODXs. In the cluster, 225 genes and 15 biological pathways were nominated, including the proteoglycans in the cancer (11 genes), cell cycle (8 genes), P53 signaling pathway (6 genes).

### Experimental Applications in OICI-EPS-0530 and OICI-EPS-0486

**Figure 6:**
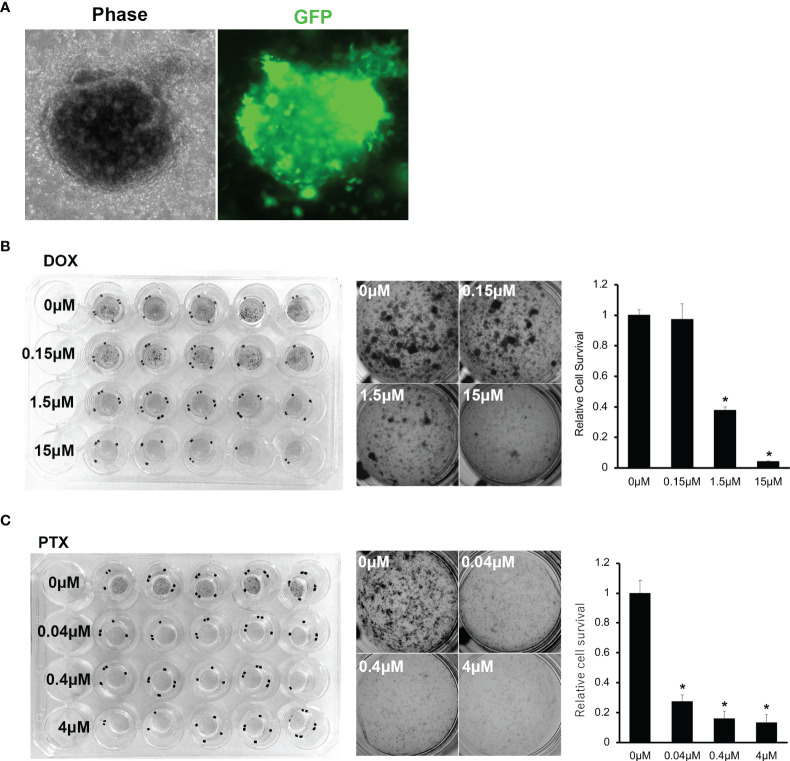
**(A)** Microscopic images of PDO of OICI-EPS-0530 expressing green fluorescent protein (GFP). Left; OICI-EPS-0530 organoids under ALI organoid culture by phase-contrast microscopy. Right; an image of the same organoid with GFP. **(B)** Organoid proliferation assay with MTT assay against Doxorubicin (DOX). Left; Image of whole culture plate. Middle; Each well by concentration of DOX. Right; Relative organoids viability by concentration of DOX. (n = 5; *P < 0.005). **(C)** Organoid proliferation assay with MTT assay against Paclitaxel (PTX). Left; Image of whole culture plate. Middle; Each well by concentration of PTX. Right; Relative organoids viability by concentration of PTX. (n = 5; *P < 0.005).

It was essential to determine whether the established EPS organoid line could be used with various experiments. Firstly, we induced GFP with lentivirus infection for OICI-EPS-0530 organoid cells and observed the re-formation of the organoids and expression of GFP in the EPS organoids (**Figure 6A**). This means that the established organoid line could be used for RNA interference (RNAi) experiments. We performed drug treatment experiments, using doxorubicin or paclitaxel for the organoid *in vitro*, and found that 1.5-15μM of doxorubicin and 0.04-4μM of paclitaxel significantly suppressed the organoid growth of OICI-EPS-0530. In addition, 1.5-15μM of doxorubicin showed similar efficacy for the organid growth and 0.04-4μM of paclitaxel moderately suppressed the organoid growth of OICI-EPS-0486. It demonstrated that OICI-EPS-0530 and OICI-EPS-0486 could be used for drug screening experiments. In addition, OICI-EPS-0530 and OICI-EPS-0486 were resistant to doxorubicin, which was the first-line drug for soft tissue sarcoma, and OICI-EPS-0530, but not OICI-EPS-0486, was sensitive to paclitaxel, which was a widely used taxane anticancer drug, according to the database of drug sensitivity and Genomics of Drug Sensitivity in Cancer (**Figures 6B, C**, **Supplementary Figures 7A, B** and **Table S7**) (35).

## Discussion

Epithelioid sarcoma is one of the most aggressive soft tissue sarcomas which is resistant to most treatments including chemotherapies and radiotherapy (16, 18). Therefore, the development of novel treatments for various malignant tumors and rare cancers including EPS using bioresources which have similar phenotypes to tumors in the human body are essential. The organoid culture method has the potential to overcome the problem mentioned above. However, most 3-dimentional culture methods used in the studies on sarcoma were spheroid culture and few reports have investigated organoids in sarcoma (30, 31). Moreover, there is no report on the establishment of an ODX model using sarcoma. Therefore, to the best of our knowledge, this is the first report on the establishment of PDO and ODX models using soft tissue sarcoma, EPS.

The global genomic profile of established EPS organoid line, OICI-EPS-0530, was investigated in this study, and we showed the moderate difference between an original EPS tumor and ODXs. The results may be owing to the various types of normal cells in the original tumor and/or change or enrichment of genetic phenotype in EPS tumor cells. In applying the concept of organoid, it was possible for the developed tumor to have the enriched phenotype of the original EPS tumor cells. In order to clarify that, more organoid lines of sarcoma (organoid panel) and further investigations are needed. Recently, a study on autophagy induction reported a resistant mechanism by cytotoxic and EZH2 inhibition in an INI1-negative EPS patient-derived xenograft (36). The expression of almost all the genes known to be related to autophagy machinery/regulation in Ref. 37 were preserved in the original tumor and ODXs of OICI-EPS-0530 (**Table S8**). In addition, OICI-EPS-0530 showed sensitivity to paclitaxel (**Figure 6C**). Recently, expression of tubulin genes, TUBB3 and TUBB6, was significantly downregulated in the taxane-resistant breast cancers (37). In OICI-EPS-0530, those gene expressions were upregulated in original EPS tumor and ODXs, so that paclitaxel might suppress the growth of EPS organoids.

In this study, we reported the establishment of novel EPS organoid lines using the ALI organoid culture method. In our impression, it was easy to handle the tumor cells *in vitro* using the ALI method than the common organoid culture method using Matrigel. Moreover, the surgically resected sarcoma tunors were often big, thus, much tumor samples could be used. We could begin the culture with much tumor cells *in vitro* with the ALI method, therefore, xenograft was easy. In contrast, some problems were encountered during culturing of the sarcoma organoids using the ALI method. Lysed collagen and inactive organoids in the primary culture or serial passages were sometimes observed. In such situations, they were quickly passaged or collagen was added on the upper layer of the collagen and the cells observed for recovering. In addition, it was difficult to decide the timing of the passages because the organoids had a 3D structure and it was often difficult to culture them. In such a case, the organoids were passaged after 1 month after the start of the culture.

At the beginning of our research, we tried to use Matrigel, which is often used in various cancers and is cultured from a single cell from several types of sarcomas, but it did not work (38–40). It might be an advantage for organoid growth to be able to begin sarcoma organoid culture from a clump of cancer cells and to use collagen. In addition, it was possible that aeration leading to cell polarity by the ALI organoid culture method was essential for sarcoma organoid culture. In order to clarify whether the ALI organoid culture method is suitable for sarcoma, an accumulation of experiences is needed. We are still to establish the other histological types of sarcomas, including liposarcoma, chondrosarcoma, synovial sarcoma, and undifferentiated pleomorphic sarcoma, and to determine the better way to culture sarcoma organoids. High-volume cancer centers play an important role in the development of useful bioresources for rare cancers including sarcoma.

The present study has several limitations. First, we just established 2 sarcoma organoid lines of EPS with ALI organoid culture method. Thus, it was uncertain whether the ALI organoid culture method was suitable for various type of sarcoma. Second, this study was performed in single institution. Therefore, it is possible that the unintentional bias in experimental procedures could not be fully eliminated. Further investigation on larger numbers of sarcoma samples is required.

In conclusion, newly established OICI-EPS-0530 and OICI-EPS-0486 organoid lines preserved the morphological and genetic characteristics of the original tumor diagnosed as EPS. Both organoids had tumorigenesis in a NSG mouse, thus they are excellent bioresources for further investigation of the pathogenesis and novel treatments of EPS. This study demonstrated that the ALI organoid culture method could be applied to the various type of sarcoma.

## Data Availability Statement

The datasets presented in this study can be found in online repositories. The names of the repository/repositories and accession number(s) can be found in the article/**Supplementary Material**.

## Ethics Statement

The studies involving human participants were reviewed and approved by Institutional Review Board for Clinical Research. The patients/participants provided their written informed consent to participate in this study. Written informed consent was obtained from the individual(s) for the publication of any potentially identifiable images or data included in this article. The animal study was reviewed and approved by Institutional committee of animal care of the Osaka International Cancer Institute.

## Author Contributions

TW, YY, SS, and ST designed the study. TW collected, interpreted, and analyzed the data. TW performed the statistical analyses. TW wrote the manuscript. TW, HO, KY, KS, and YM performed *in vitro* and mouse experiments. HT, TW, YI, SoN, TY, and ST treated the patients. ShN performed the pathological diagnosis and experiments. All the authors revised the manuscript critically for important intellectual content and read and approved the final manuscript.

## Funding

The present study was supported by JSPS KAKENHI (grant no. JP20K18048).

## Conflict of Interest

The authors declare that the research was conducted in the absence of any commercial or financial relationships that could be construed as a potential conflict of interest.

## Publisher’s Note

All claims expressed in this article are solely those of the authors and do not necessarily represent those of their affiliated organizations, or those of the publisher, the editors and the reviewers. Any product that may be evaluated in this article, or claim that may be made by its manufacturer, is not guaranteed or endorsed by the publisher.

## Abbrevriations

PDO, Patient derived organoid ; ODX, Organoid derived xenograft; ALI, Air liquid interface; EPS,Epithelioid sarcoma; 3D, 3-dimentional; NSG, NOD-scid IL2Rgnull ; INI-1, Integrase interactor 1; CDF, Continuous disease-free; PBS, Phosphate-buffered saline; NGS, next generation sequencing.
